# A Rapid Motor Task-Based Screening Tool for Parkinsonism in Community-Based Studies

**DOI:** 10.3389/fneur.2021.653066

**Published:** 2021-05-13

**Authors:** Wendy W. Dlamini, Searles Nielsen, Mwiza Ushe, Gill Nelson, Brad A. Racette

**Affiliations:** ^1^Department of Neurology, Washington University School of Medicine, St. Louis, MO, United States; ^2^Faculty of Health Sciences, School of Public Health, University of the Witwatersrand, Parktown, Johannesburg, South Africa; ^3^Institute for Global Health, University College London, London, United Kingdom

**Keywords:** kinematics, manganese, parkinsonism, predictive model, receiver operating characteristic

## Abstract

**Background:** The prevalence of parkinsonism in developing countries is largely unknown due to difficulty in ascertainment because access to neurologists is often limited.

**Objective:** Develop and validate a parkinsonism screening tool using objective motor task-based tests that can be administered by non-clinicians.

**Methods:** In a cross-sectional population-based sample from South Africa, we evaluated 315 adults, age >40, from an Mn-exposed (smelter) community, using the Unified Parkinson Disease Rating Scale motor subsection 3 (UPDRS3), Purdue grooved pegboard, and kinematic-UPDRS3-based motor tasks. In 275 participants (training dataset), we constructed a linear regression model to predict UPDRS3. We selected motor task summary measures independently associated with UPDRS3 (*p* < 0.05). We validated the model internally in the remaining 40 participants from the manganese-exposed community (test dataset) using the area under the receiver operating characteristic curve (AUC), and externally in another population-based sample of 90 participants from another South African community with only background levels of environmental Mn exposure.

**Results:** The mean UPDRS3 score in participants from the Mn-exposed community was 9.1 in both the training and test datasets (standard deviation = 6.4 and 6.1, respectively). Together, 57 (18.1%) participants in this community had a UPDRS3 ≥ 15, including three with Parkinson's disease. In the non-exposed community, the mean UPDRS3 was 3.9 (standard deviation = 4.3). Three (3.3%) had a UPDRS3 ≥ 15. Grooved pegboard time and mean velocity for hand rotation and finger tapping tasks were strongly associated with UPDRS3. Using these motor task summary measures and age, the UPDRS3 predictive model performed very well. In the test dataset, AUCs were 0.81 (95% CI 0.68, 0.94) and 0.91 (95% CI 0.81, 1.00) for cut points for neurologist-assessed UPDRS3 ≥ 10 and UPDRS3 ≥ 15, respectively. In the external validation dataset, the AUC was 0.85 (95% CI 0.73, 0.97) for UPDRS3 ≥ 10. AUCs were 0.76–0.82 when excluding age.

**Conclusion:** A predictive model based on a series of objective motor tasks performs very well in assessing severity of parkinsonism in both Mn-exposed and non-exposed population-based cohorts.

## Introduction

The Global Burden of Disease Study estimates that the number of people affected by Parkinson's disease (PD) more than doubled from 1990 to 2015 with the highest prevalence in high-income regions and lowest in sub-Saharan Africa and Eastern Europe in 2015 (1, 2). However, little is known about the burden of the disease in resource-poor environments such as many regions in Africa (3). Relatively low reported PD prevalence in Africa is almost certainly inaccurate, since case identification is challenging in countries that lack sufficient research and clinical expertise to survey their populations (4). Globally, there are 3.1 neurologists per 100,000 people, whereas in Africa there are only 0.1 neurologists per 100,000 people (5). Given the increasing life expectancy in many African countries, estimating true disease burden of diseases of aging, such as PD, is critical to providing adequate healthcare resources for these patients.

The Unified Parkinson Disease Rating Scale motor subsection 3 (UPDRS3) remains the most widely used tool to quantify parkinsonian motor signs in patient and non-patient populations (6–10). In addition to quantifying parkinsonism severity, this standardized examination elicits the cardinal signs required to make a diagnosis of PD. Nevertheless, many developing countries lack the relevant clinical expertise required to quantify parkinsonism or diagnose PD accurately. For this study, we sought to develop a motor battery that can be used to predict UPDRS3 scores in population-based African cohorts. In practice, such estimates of the UPDRS3 score might be useful for initial screening to identify those who should receive further evaluation by a neurologist. These UPDRS3 estimates might also be suitable for epidemiological studies investigating neurological health effects of environmental or occupational exposures when UPDRS3 assessment by a movement disorders specialist is not feasible.

## Materials and Methods

### Standard Protocol Approvals

The Washington University School of Medicine Human Research Protection Office (St. Louis, Missouri, United States) and the University of the Witwatersrand Human Research Ethics Committee (Johannesburg, Gauteng, South Africa) approved this study. All participants provided written informed consent.

### Participants

Within a cross-sectional population-based study with 315 South African adults age >40 and living <5 km from a large manganese (Mn) smelter in Meyerton, South Africa, we developed and validated a predictive model for UPDRS3, in training (*N* = 275, 87%) and test (*N* = 40, 13%) datasets, respectively. We enrolled the participants in these two groups consecutively (Supplementary Figure 1). Participants in the training dataset lived a mean of 1.85 km (*SD* = 0.77) from the smelter, and the participants in the test dataset lived a mean of 1.96 Km (*SD* = 0.74) from the smelter. The participants from Meyerton comprised a subset of participants who were recruited as part of a larger environmental Mn study (11). The recruitment approach for this larger study was based upon the location of residence, and was designed to obtain a true population-based sample within three Meyerton-based settlements. Briefly, we pre-selected every other residence (two communities) or all residences (one, smaller community). We attempted to recruit all age-eligible adults in each pre-selected residence to participate in the study, or, in the two communities where only half of residences were selected, the residence to the left if no one was home or eligible. Across the three settlements in the Meyerton community, 462/666 (69.4%) of homes that we visited had at least one eligible adult who agreed to participate. Air monitoring in all three Meyerton settlements confirmed relatively high mean concentrations of PM_2.5_-Mn (203 ng/m^3^ at a long-term fixed site in one settlement, and based upon concurrent sampling approximately half that level in the other two settlements) (11). This is ~12–20 times higher than mean PM_2.5_-Mn in other populated areas in South Africa (11, 12), and ~4 times higher than the mean modeled PM_2.5_-Mn exposure levels for an Mn smelter community in the United States (13). We therefore refer to all participants from this community as Mn-exposed.

We externally validated our model in 90 additional participants, also >40 years old, from a community in the same province, Ethembalethu. Ethembalethu was smaller than the Meyerton-based settlements, so we attempted to recruit every age-eligible resident using the same door-to-door approach to obtain a population-based sample. In this community, 79/108 (73.1%) of homes that we visited had at least one age-eligible adult who agreed to participate. Ethembalethu is an industry-free community, with no nearby Mn smelting or mining operations, located ~70 km from Meyerton. Air monitoring in Ethembalethu demonstrated mean concentrations of PM_2.5_-Mn ~20 times lower than at the fixed site in Meyerton (10 ng/m^3^) (11). This is ~40% lower than mean PM_2.5_-Mn levels measured in the air in a city on the southeastern coast of South Africa with some industry but no Mn smelting or mining activities (12). We therefore refer to all participants from Ethembalethu as non-exposed.

For inclusion in the present work, we required participants in both communities to meet the following criteria: (1) be non-ambidextrous (self-report as right-handed or left-handed), (2) complete at least one trial for the dominant and non-dominant hand for each of five motor tasks under the direction of a trained test administrator, (3) have been examined by a movement disorder specialist using the UPDRS3 and had complete UPDRS3 data. Exclusion criteria were: (1) current use of neuroleptic medications, and (2) neurologic co-morbidities that might compromise the accuracy of the UPDRS3, such as stroke or spasticity.

In order to include individuals with other conditions that would cause incomplete UPDRS3 data, such as injured/missing limbs or inability to undergo the pull test to assess balance, we used imputation of the respective subscore(s) to obtain complete UPDRS3 data when possible. We included individuals regardless of UPDRS3 score, presence of PD, or occupational Mn exposure, in order to ensure that our original population-based samples remained representative of the respective settlements. The above exclusions, need for imputation, presence of PD, and past or current occupational Mn exposure were all uncommon (all <2.5%) in the larger study (11).

All recruitment, testing, and examination occurred in 2016–2020 (11).

### Grooved Pegboard and Kinematic Testing

Participants completed five motor tasks—the grooved pegboard task and four accelerometry-based kinematic-UPDRS3 tasks—in their homes, at the time of enrollment into the original study. These kinematic-UPDRS3 tasks were designed to characterize finger tapping, hand rotation, action tremor, and postural tremor. All participants had grooved pegboard and kinematic testing conducted by one of three non-clinician test administrators. We trained all three test administrators previously using a video-based training module, followed by supervised administration of the tests to non-research participants. In addition, we made frequent data quality checks throughout the study. For the grooved pegboard task, we used a standard grooved pegboard device (Lafayette Instrument Company, Lafayette, Indiana) and followed published testing procedures (14). For the four kinematic tests, test administrators placed a wireless motion sensor (Kinesia^TM^, Great Lakes NeuroTechnologies, Independence, Ohio) (15–19) on the top of the participant's index finger. The Kinesia Motion Sensory device comprises a triaxial accelerometer and triaxial gyroscope, allowing measurement of acceleration (linear) and velocity (angular), respectively, along all three axes (x, y, and z) at 64 Hertz. We recorded the digitized signals on a computer tablet, using motion capture software (Great Lakes NeuroTechnologies, Independence, Ohio), following at least one non-recorded practice trial. Participants then completed three 12-second trials while seated for each hand for each task: (1) *postural tremor*—participant was instructed to raise both arms, straight out in front of his/her body and stay as still as possible; (2) *action tremor*—participant alternated touching his/her index finger to his/her nose and to the administrator's finger held an arm's length away from the participant; (3) *finger tapping*—participant tapped his/her index finger and thumb together while keeping the other fingers stable and the elbow extended; (4) *hand rotation*—participant rotated his/her hand at the wrist, positioning the arm so that the elbow was flexed and the hand open. Participants were instructed to perform finger tapping and hand rotation tasks with as large an amplitude and as fast, as possible. Participants completed the trials for the right hand first, followed by the left hand, for each of these four tasks. A previous study in PD patients that used a similar Kinesia Motion Sensory device found that in the more parkinsonian hand the test-retest reliability across three 15-s trials, as measured by the intraclass correlation coefficient, was 0.71 for postural tremor and 0.94 for finger tapping speed (18).

We then developed, validated, and applied comprehensive computer code to process these large datasets (Supplementary Material 1). Specifically, we first checked and standardized data from each trial. For example, we removed kinematic data for trials that appeared to be incomplete (<12 s) or for sensor failures. We then calculated six summary measures across the three trials (mean velocity, mean peak velocity, coefficient of variation, decrement in peak velocity, cycles/second, and decrement in cycles/second; Supplementary Table 1). We also calculated three summary measures for each hand from grooved pegboard testing (time to place all 25 pegs, number of pegs placed, number of pegs dropped). We calculated all of the kinematic and grooved pegboard measures for each hand (dominant, non-dominant), using a mean of all ≤ 3 trials and by taking only the first trial. As an additional variation for mean velocity for the finger tapping and hand rotation tasks, we isolated both the upward/downward motions and the clockwise/counterclockwise motions, respectively.

### Assessment of UPDRS3 Score and Subscores

One movement disorder specialist (BR) examined all participants using the UPDRS3 (20), blinded to performance on the grooved pegboard and kinematic motor tasks. The examination occurred in one central non-clinical location in each of the two communities, while study staff conducted the testing in-home on an earlier date (a median of 37 and 3 days earlier, respectively, for Meyerton and Ethembalethu) without the examiner present, and individual testing results were not available to the examiner. In addition to UPDRS3 total score, we focused on selected UPDRS3 subscores (upper limb bradykinesia and tremor) to facilitate validation and selection of grooved pegboard and kinematic summary measures for development of the prediction of the total UPDRS3 score (Supplementary Material 2).

### Determination of Handedness and Demographic Variables

We used self-reported handedness to classify UPDRS3 subscores and the five motor tasks as dominant or non-dominant. Participants also provided socio-demographic information, including age, sex, ethnicity, and home language.

### Statistical Analysis

We performed all data processing and statistical analyses using Stata version MP 14.2 (StataCorp, College Station, Texas) (21). To help prioritize which summary measures to include in the predictive model, we estimated Spearman's ρ correlation coefficients between each of the kinematic and grooved pegboard test summary measures and the respective UPDRS3 subscores (our gold standard) (Supplementary Material 2). Specifically, we sought to identify summary measures with the Spearman's ρ correlation coefficients of the greatest magnitude (relative to each other, or at least weakly or significantly correlated, i.e., ρ > 0.20 and/or with *p* < 0.05) and, more importantly, in the expected direction (positive or negative). Greater UPDRS3 scores and subscores indicate greater parkinsonism, which we anticipated would be associated with longer grooved pegboard times, fewer pegs placed, and more pegs dropped. With regard to the kinematic tests, we also anticipated that greater UPDRS3 scores would be associated with lower velocities (or fewer cycles per second) and greater decrement (and hence greater variability as assessed by the coefficient of variation) on the finger tapping and hand rotation tasks. We investigated whether correlations differed according to hand dominance and whether motor task data from only one hand might be sufficient to predict UPDRS3. We also examined the association between summary measures derived from only the first of the three trials to determine if a single trial would be sufficient for UPDRS3 prediction.

### Model Development

We used linear regression with the total neurologist rated UPDRS3 as the outcome variable, to predict UPDRS3 in our training dataset (*N* = 275). Age and motor task summary measures selected above were our primary *a priori* predictors of interest, and we initially retained all as continuous measures (8, 22). We then used locally weighted scatterplot-smoothing (LOWESS) graphs to determine, for each of these predictors, whether linear modeling or other approaches were most appropriate. The LOWESS graphs suggested a quadratic term for hand rotation and finger tapping mean velocities, which we verified and confirmed to be true for only hand rotation (In a simple linear regression between UPDRS3, hand rotation mean velocity, and its quadratic term, the quadratic term was statistically significant; therefore, we included this term in our model. In contrast, the square term for finger tapping was not statistically significant when including finger tapping mean velocity). To assess multicollinearity, we used the variance inflation factor (VIF) and conservatively verified that the VIF was <2 for all predictors in our final model. The exception was the hand rotation linear term and its quadratic term because these are inherently correlated, so both were included to better capture the true association between that predictor variable and UPDRS3. Secondarily, we repeated this model development process but did not allow age to be included as a predictor, given that age in the Mn-exposed community could be a surrogate for duration of Mn exposure, and therefore the coefficient for age might not translate well to settings without this potential cause of parkinsonism.

### Model Validation

We formally validated model performance in our test dataset (*N* = 40) and in the independent, external dataset (*N* = 90), using the receiver operating characteristic (ROC) curve (21, 23). We estimated the area under the ROC curve (AUC) using a dichotomized UPDRS3 variable (UPDRS3 ≥ 10 and UPDRS3 ≥ 15, when possible) as our gold standard. We used a UPDRS3 score of 15 as a cut point because most idiopathic PD patients become symptomatic and present for medical attention with UPDRS3 scores ≥15 (24–26), i.e., that this threshold would reflect functionally impairing motor dysfunction. We also used a UPDRS3 score of 10 as a cut point because our primary focus was to develop a screening tool, i.e., one would likely use a more conservative (lower) UPDRS3 than 15. We also calculated sensitivity and specificity for these same gold standard variables at selected predicted UPDRS3 cut points. In order to obtain an overall measure of whether the predicted UPDRS3 might be suitable for use as a continuous outcome measure in epidemiologic studies, we calculated Spearman's ρ correlation coefficient to measure agreement between neurologist-assessed UPDRS3 and the model-derived UPDRS3. We calculated the 95% confidence interval (CI) for this ρ and for the AUCs. Obtaining an estimate for the lower CI is more informative than a *p*-value. A CI for ρ that excludes zero indicates significance at a two-sided α = 0.05, but for this particular comparison we viewed a lower CI > 0.20 to indicate clearly that the observed correlation was at least weakly positive. An AUC of 0.5 indicates discrimination ability no better than chance, an AUC of 0.7 is fair, an AUC of 0.8 is good, an AUC of 0.9 is excellent, and an AUC of 1.0 indicates perfect discrimination (27). Therefore, we viewed a lower CI > 0.70 to indicate clearly that the observed AUC was at least fair.

## Results

### Characteristics of Participants

Most (98.5%) of participants were of black/African ethnicity, with a median age of 51 (Table 1). The mean UPDRS3 score was 7.9 (*SD* = 6.3, range 0.0–38.5), but differed markedly between the two communities. Upper limb bradykinesia contributed substantially to UPDRS3 as 16.8% of participants had >6 points in total across the six upper limb bradykinesia subscores, i.e., a subscore of >1 on at least one of these six scores (not shown). In contrast, action/postural tremor was relatively uncommon. Only 20 (4.9%) of the participants had an action/postural tremor subscore of 2, and only three participants had prominent action/postural tremor or only rest tremor (subscore >2). Three participants who had complete kinematic and grooved pegboard data, all from Meyerton, had PD according to this neurological examination.

**Table 1:** Participant characteristics, overall and by environmental manganese (Mn) exposure status, South Africa, 2016–2020.

		**Mn-exposed** ** community** ** (Meyerton)**		**Non-exposed community (Ethembalethu)**
	**All participants** ***N* = 405** ***n* (%)**	**Training dataset** ***N* = 275** ***n* (%)**	**Test (internal validation) dataset** ***N* = 40**	**External validation dataset *N* = 90**
**Characteristic**			***n* (%)**	***n* (%)**
Sex				
Male	160 (39.5)	122 (44.4)	10 (25.0)	28 (31.1)
Female	245 (60.5)	153 (55.6)	30 (75.0)	62 (68.9)
Black/African^a^	398 (98.5)	271 (98.6)	40 (100.0)	87 (97.8)
Language^b^				
Sesotho	194 (48.1)	155 (56.8)	26 (65.0)	13 (14.4)
IsiXhosa	57 (14.1)	47 (17.2)	4 (10.0)	6 (6.7)
IsiZulu	60 (14.9)	40 (14.7)	4 (10.0)	16 (17.8)
Other^c^	92 (22.8)	31 (11.4)	6 (15.0)	55 (61.1)
Education^d^				
None/non-formal schooling	58 (14.3)	38 (13.8)	11 (27.5)	9 (10.0)
Primary	150 (37.0)	94 (34.2)	18 (45.0)	38 (42.2)
Secondary	130 (32.1)	96 (34.9)	6 (15.0)	28 (31.1)
Matric or higher	67 (16.5)	47 (17.1)	5 (12.5)	15 (16.7)
Ever Mn occupational exposure	10 (2.5)	10 (3.6)	0 (0.0)	0 (0.0)
Current Mn occupational exposure	2 (0.5)	2 (0.7)	0 (0.0)	0 (0.0)
UPDRS3 ≥ 10	146 (36.1)	119 (43.3)	20 (50.0)	7 (7.8)
UPDRS3 ≥ 15	60 (14.8)	51 (18.6)	6 (15.0)	3 (3.3)
**Characteristic**	**Mean (*****SD*****)**	**Mean (*****SD*****)**	**Mean (*****SD*****)**	**Mean (*****SD*****)**
Age, years	52.4 (8.7)	51.3 (8.2)	53.2 (9.8)	55.6 (8.6)
Minimum	41	41	41	41
25th percentile	45	45	44.5	51
Median	51	50	51.5	55
75th percentile	57	56	60	59
Maximum	84	81	79	84
Distance from Mn smelter, km	–^e^	1.85 (0.77)	1.96 (0.74)	–^e^
Minimum	–^e^	0.83	0.97	–^e^
25th percentile	–^e^	1.22	1.21	–^e^
Median	–^e^	1.54	2.10	–^e^
75th percentile	–^e^	2.80	2.51	–^e^
Maximum	–^e^	3.15	3.15	–^e^
UPDRS3	7.9 (6.3)	9.1 (6.4)	9.1 (6.1)	3.9 (4.3)
Minimum	0.0	0.0	0.0	0.0
25th percentile	2.5	4.0	4.0	1.0
Median	7.0	8.0	9.5	2.0
75th percentile	12.0	13.0	13.0	5.5
Maximum	38.5	38.5	29.5	21.5
Non-dominant hand grooved pegboard time, seconds^f^	119.7 (48.5)	116.2 (47.6)	139.5 (56.2)	121.7 (45.5)
Minimum	51.4	51.4	64.9	60.0
25th percentile	85.2	85.0	93.8	85.1
Median	106.0	103.8	121.5	113.5
75th percentile	135.7	129.9	182.4	151.7
Maximum	300.0	300.0	300.0	241.1
Non-dominant hand rotation, mean velocity^g^	597.1 (164.8)	614.2 (177.0)	574.2 (174.5)	583.5 (163.7)
Minimum	233.9	151.7	280.4	236.9
25th percentile	480.0	487.6	452.1	449.7
Median	594.0	610.9	516.1	587.6
75th percentile	699.8	729.7	696.8	686.9
Maximum	1,257.7	1,257.7	1,013.3	975.9
Non-dominant finger tapping, mean velocity	352.5 (118.1)	352.0 (115.6)	395.8 (162.1)	408.2 (124.5)
Minimum	87.8	95.5	138.7	168.5
25th percentile	269.0	268.8	282.3	316.3
Median	343.5	347.3	395.5	413.2
75th percentile	426.3	432.0	470.2	507.5
Maximum	727.0	699.0	727.0	679.1

*SD, standard deviation; UPDRS3, Unified Parkinson's Disease Rating Scale motor subsection 3; Mn, manganese; km, kilometer*.

a *Total and external validation dataset percent excludes 1 participant with missing data for race. Other 6 participants were white or of mixed race*.

b *Total and training dataset percent excludes 2 participants with missing data on language*.

c *Setswana, Sepedi, Xitsonga, Afrikaans, SiSwati, and Tshivenda*.

d *Primary is grades 1–7, secondary is grades 8–11, and matric is grade 12*.

e *Only shown for participants from the Mn smelter community of Meyerton; the non-exposed community of Ethembalethu is >70 km from Meyerton*.

f *Total and external validation dataset excludes 2 participants with missing data for this motor task*.

g *All available kinematic trials, except for the training dataset, in which only the first kinematic trial was used for development of the UPDRS3 predictive model, in order to minimize the time required to conduct screening*.

### Performance of Summary Measures

Several summary measures for upper limb bradykinesia correlated with the respective UPDRS3 subscores (Supplementary Table 2), with grooved pegboard time and hand rotation and finger tapping mean velocities demonstrating the greatest agreement. Of these three measures, the mean velocity for hand rotation demonstrated the best agreement (ρ = −0.43 and ρ = −0.42 for the dominant and non-dominant hand, respectively), with the corresponding UPDRS3 subscore, i.e., rapid alternating movements. Agreement for these kinematic summary measures was not improved by isolating the direction of the movement, i.e., upward vs. downward motions within finger tapping or clockwise vs. counterclockwise motions within rapid alternating movements. The other kinematic summary measures (cycles/second, decrement in peak velocity, and decrement in cycles/second) did not perform as well as mean and peak velocities as a measure of upper limb bradykinesia. Of these, the greatest agreement was for coefficient of variation and “decrement” in the non-dominant hand for the hand rotation task (Supplementary Table 2).

The absolute value of the correlation coefficients between the action/postural tremor summary measures and UPDRS3 subscore were all well below 0.20 (action tremor task: ρ = −0.06 to 0.08; postural tremor task: ρ = −0.11 to 0.06; Supplementary Table 3).

When comparing a given measure to the respective UPDRS3 subscore, dominant and non-dominant hands yielded similar Spearman's ρ correlation coefficients (Supplementary Table 2). However, testing from the non-dominant hand performed slightly better for the “decrement” summary measures. We found similar correlations between UPDRS3 and kinematic testing when we used only the first trial, rather than the mean of all available trials (Supplementary Table 4).

### UPDRS3 Predictive Model

**Figure 1:**
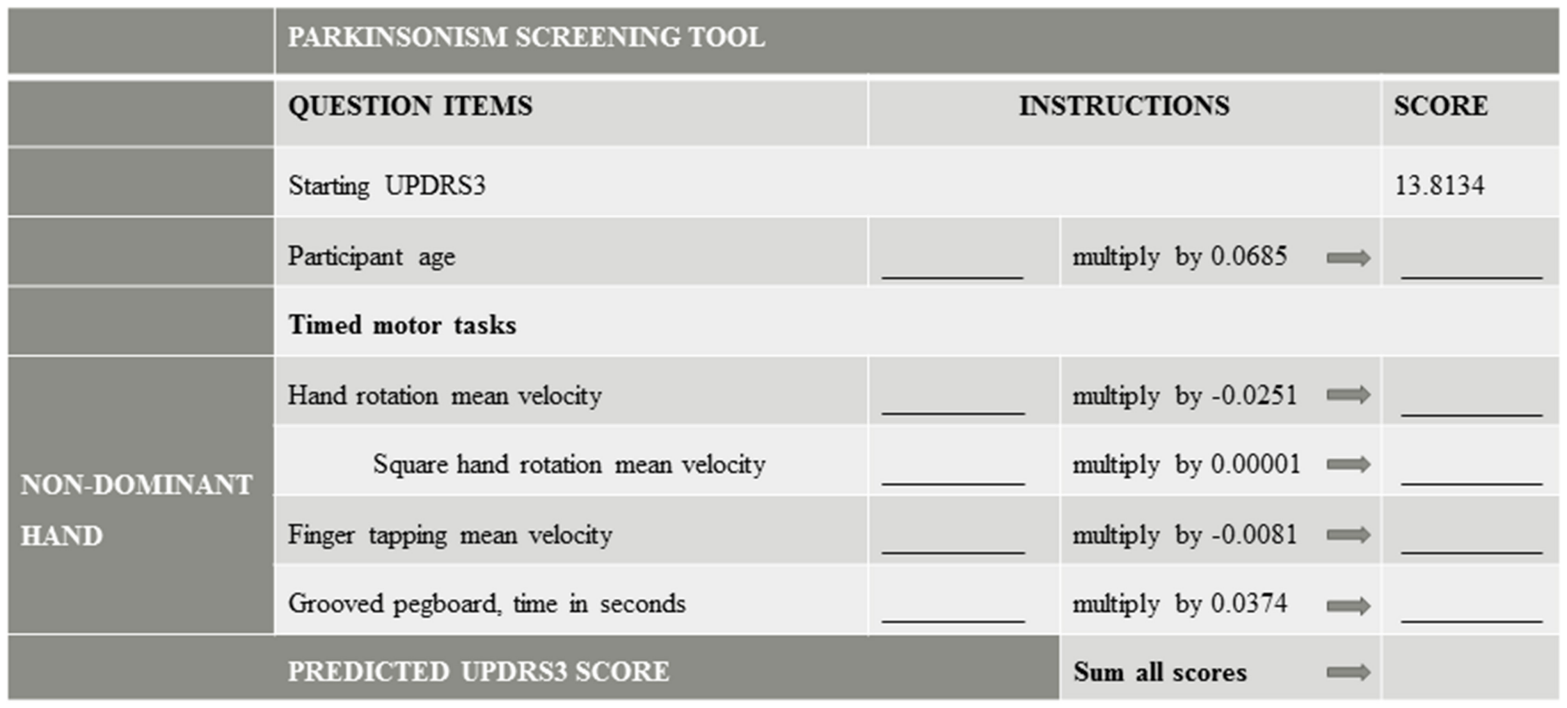
Parkinsonism Screening Tool. A simple motor task screening tool based on the predictive model, which is shown in detail in Table 2. UPDRS3, Unified Parkinson's Disease Rating Scale motor subsection 3.

The final model included the following predictors for one trial from the non-dominant hand: hand rotation kinematic mean velocity linear term and quadratic term (squared term); finger tapping kinematic mean velocity; grooved pegboard time; and age, each as linear terms (Table 2, Figure 1). Finger tapping and hand rotation mean velocities were inversely associated with UPDRS3, whereas age and grooved pegboard time were positively associated with UPDRS3. Administration of the tests selected for this screening tool for this predictive model took <10 min (Figure 1).

**Table 2:** UPDRS3 predictive model in the training dataset (*N* = 275), South Africa, 2016–2020.

	**Unadjusted**			**Mutually adjusted**^**a**^ **(final predictive model)**		
**Characteristic**	**Linear regression **β**^b^**	**95% CI**	***p*-value**	**Linear regression **β**^b^**	**95% CI**	***p*-value**
Motor tasks (non-dominant hand)						
Hand rotation mean velocity^c^	−0.0395	−0.06, −0.02	<0.001	−0.0251	−0.04, −0.01	0.01
Hand rotation mean velocity squared^c^	0.00002	0.00001, 0.00004	0.01	0.00001	−0.000001, 0.00003	0.08
Finger tapping mean velocity^c^	−0.0162	−0.02, −0.01	<0.001	−0.0081	−0.01, −0.002	0.01
Grooved pegboard time, seconds	0.0547	0.04, 0.07	<0.001	0.0374	0.02, 0.05	<0.001
Age, years	0.2177	0.13, 0.31	<0.001	0.0685	−0.02, 0.16	0.14
Constant	–^d^	–^d^	–^d^	13.8134	5.64, 21.99	0.001

*CI, confidence interval; UPDRS3, Unified Parkinson's Disease Rating Scale motor subsection 3*.

a *Mutually adjusted indicates that all estimates are adjusted for all listed variables. When excluding age (non-significant) the constant was 17.0251, the coefficients were as follows: −0.0245 for hand rotation, 0.00001 for hand rotation squared, −0.0093 for finger tapping, and 0.0421 for grooved pegboard*.

b *Difference in UPDRS3 score per unit change in the motor tasks and age*.

c *First kinematic trial*.

d *Differs for each model*.

### Model Performance

**Figure 2:**
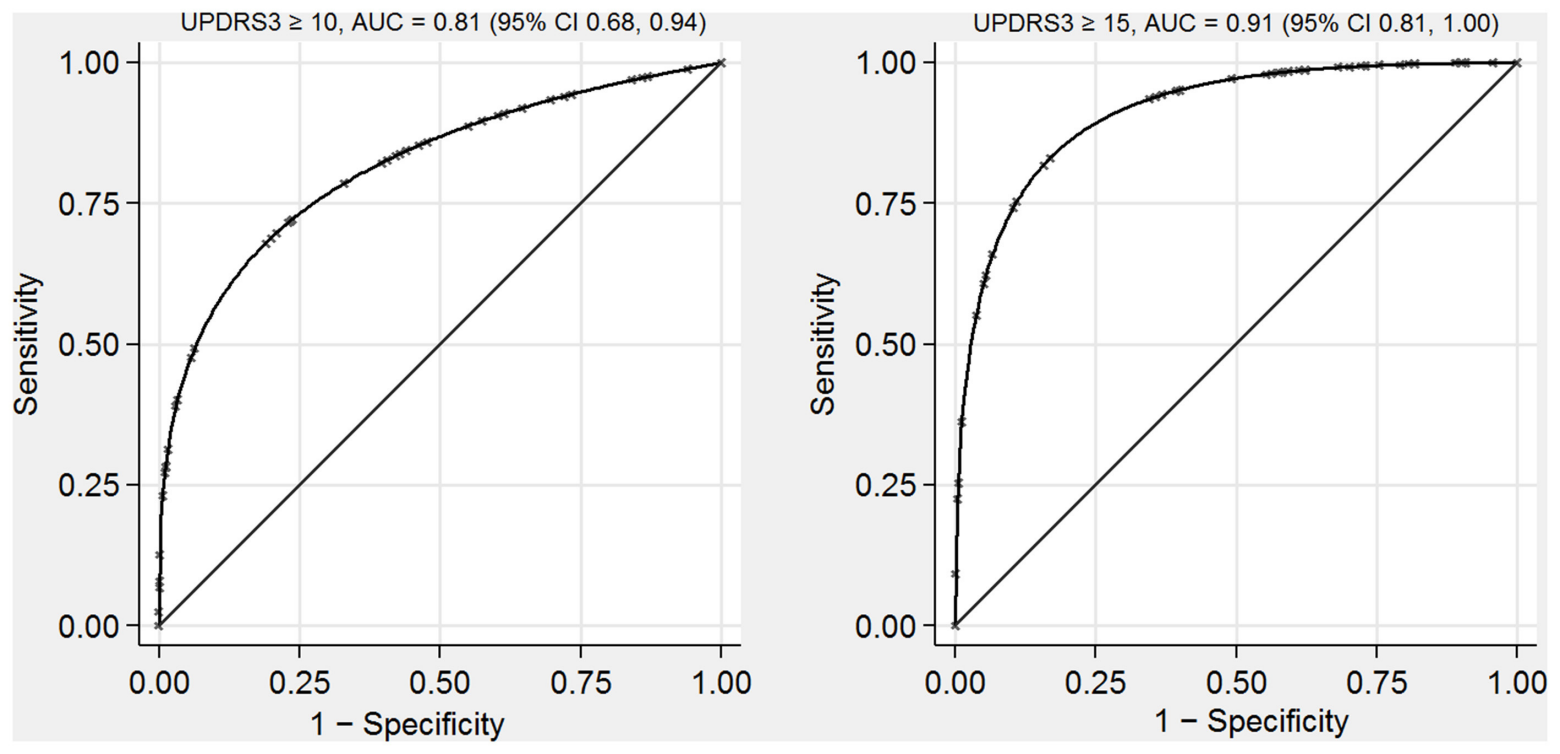
Receiver operating characteristic curves for identifying individuals with UPDRS3 ≥ 10 and UPDRS3 ≥ 15 in a population-based sample with environmental Mn exposure, South Africa, 2016–2020. The AUCs indicate that the UPDRS3 predictive model performed well. AUC, area under the receiver operating characteristic curve; UPDRS3, Unified Parkinson's Disease Rating Scale motor subsection 3; Mn, manganese.

In our test dataset, performance of the predictive model as measured by the AUC was 0.81 (95% CI 0.68, 0.94) for identifying participants with neurologist-assessed UPDRS3 ≥ 10 (Figure 2). The model performed even better for identifying participants with UPDRS3 ≥ 15 (AUC = 0.91, 95% CI 0.81, 1.00). When identifying participants with UPDRS3 ≥ 10, sensitivity and specificity were equal at 70.0%, when applying a cut point of 8 for the predicted UPDRS3 score (Supplementary Table 5). Sensitivity (83.3%) and specificity (79.4%) were relatively similar for identifying participants with UPDRS3 ≥ 15 when using 11 as the cut point for the predicted UPDRS3 score. With 9 as the cut point, the model identified all participants with a UPDRS3 ≥ 15 (100% sensitivity) while allowing for a specificity of 67.7%. When retaining the predicted UPDRS3 score as a continuous measure, agreement as measured by Spearman's ρ between the neurologist-assessed UPDRS3 and predicted UPDRS3 was 0.67 (95% CI 0.48, 0.87).

When we applied our predictive model to the external validation dataset, the AUC was 0.85 (95% CI 0.73, 0.97) when identifying participants with UPDRS3 ≥ 10 (Supplementary Figure 2). Too few participants in this sample had a UPDRS3 ≥ 15 to construct a smooth ROC curve. When attempting to identify participants who had a UPDRS3 ≥ 10, a cut point of predicted UPDRS3 of 4 resulted in sensitivity of 83.3% and specificity of 68.3% (Supplementary Table 6). Agreement between the neurologist-assessed and predicted UPDRS3 as continuous variables was 0.58 (95% CI 0.46, 0.74).

When we explored the effect of using only the motor tasks as predictors, i.e., removing age from the model, AUCs were only modestly attenuated. In our test dataset the AUC was 0.76 (95% CI 0.60, 0.91) for UPDRS3 ≥ 10 and 0.86 (95% CI 0.72, 1.00) for UPDRS3 ≥ 15. In the external validation dataset the AUC was 0.82 (95% CI 0.67, 0.98).

In a *post-hoc* exploratory analysis in which we considered how well the predictive model might identify the three individuals with PD in Meyerton (training and test datasets combined), the predicted UPDRS3 scores were 16.3, 17.1, and 21.7, all above the UPDRS3 ≥ 15 cut point.

## Discussion

In this large study in South Africa we predicted the UPDRS3 score with a limited battery of kinematic and grooved pegboard tests administered by trained non-clinician community members in <10 min per participant. AUCs in two validation datasets demonstrated that performance of this predictive model for identifying individuals with UPDRS3 scores ≥10 or ≥15 was good to excellent. In addition, we confirmed that there was at least a moderate correlation between the predicted UPDRS3 scores and neurologist-assessed UPDRS3. Notably, this UPDRS3 predictive model worked equally well in communities with and without environmental Mn exposure, i.e., in population-based samples with either relatively high or relatively low UPDRS3 scores. Taken together, our findings indicate that this UPDRS3 predictive model likely can be applied to facilitate screening programs or research studies in a wide variety of settings, including under-resourced environments with limited access to PD specialists or other neurologists.

In all potential applications of this UPDRS3 predictive model, the first step after administering the three-test battery is to calculate the mean velocities from the two kinematic tests. Of the many kinematic summary measures we assessed, the mean velocities are the easiest to calculate, further ensuring the potential usefulness of our model in many situations. Calculation of these summary measures only requires the isolation of the kinematic data from the relevant axis, i.e., x-axis for finger tapping and y-axis for hand rotations, before taking the mean across all sampled time points with the respective movement. In the second step, these two means from kinematic testing, along with the grooved pegboard time and age, are simply inserted into the equation produced by our predictive model. The resulting UPDRS3 score could be used in an epidemiologic study in which it is not possible for a neurologist to examine any participants. Alternatively, in a third step, one would dichotomize the predicted UPDRS3 score according to a pre-selected cut point to identify individuals with a particular UPDRS3, corresponding to the desired sensitivity and specificity. One likely would dichotomize the predicted UPDRS3 scores at the lowest possible cut point so as to achieve the highest sensitivity that is feasible, given that a specialist would need to examine all individuals with predicted UPDRS3 scores above the selected cut point. As part of a tiered screening protocol, maximizing sensitivity would either facilitate clinical care to the largest percentage of people in a community who might benefit from treatment, or alternatively would minimize misclassification of research study participants in terms of a dichotomous outcome of interest. As evidence of the potential utility of this type of protocol, a similar tiered screening approach was adopted in a previous occupational Mn exposure setting in South Africa (28).

We acknowledge some limitations that could affect usefulness of our predictive model. First, the kinematic methods may not be easily incorporated in all settings. We trained our fieldworkers extensively in the use of kinematic devices, and we view this training as essential. Second, kinematic testing generates large amounts of data, requiring expertise in data handling. In addition, a substantial amount of computer storage and computational power would be required to process these data for many individuals at once, which might be a challenge in under-resourced environments. Third, while we used a common motion capture device, such devices have unique operational principles, instrumentation, and type of data produced (29) so data from other devices might not be ideal for use in our model. Finally, while a UPDRS3 score from a predictive model might not be as accurate as neurologist assessment, our tool is more objective than the UPDRS3. As a result, our model can address potential important challenges that might arise in epidemiologic studies, such as examiner blinding.

The strengths of our study include the use of one movement disorder specialist to obtain gold standard UPDRS3 ratings, the relatively large sample size, and a unique study population at risk for parkinsonism paired with a lower risk population, each representative of their underlying communities. These strengths positioned us well to develop and validate the resulting UPDRS3 predictive model, which also has several strengths. First, we were successful in minimizing the time required to complete the included motor testing without materially affecting performance of the UPDRS3 predictive model. Most notably, we confirmed that it was sufficient to conduct only one trial of the selected tests in one hand. Second, all predictors can be assessed objectively, i.e., without subjective assessments that might require clinical expertise. Although some prior predictive models of UPDRS3 score or “parkinsonism” benefitted from the use of objective motor assessment (either a different fine motor task or gait assessment), some of these models required test administrators to make clinical judgements (30, 31). Third, no translation of questions or questionnaires is required beyond obtaining age. Some prior predictive models required assessment of selected medical conditions (30, 31), as might be done via questioning, or even administration of full questionnaires (32). Translation burden (33), and population literacy makes a purely questionnaire-based screening protocol especially challenging to administer in low-resourced countries. Avoiding the need for translation is especially beneficial in the context of Africa and other locales in which many languages are spoken.

Restriction of our model to age and a modest battery of objective motor tasks to avoid the above potential challenges did not come at the expense of performance. Previous occupational or community-based cohorts in which investigators attempted to identify people with parkinsonism achieved AUCs ranging from 0.72 to 0.79 (30–32). The predictive model we present achieved slightly better AUCs than in these studies. Both the grooved pegboard test and the finger tapping task, such as assessed in a similar manner as here, have been shown to provide good discrimination between existing PD patients and comparable controls previously, with AUCs as high as 0.80–0.87 (34, 35). While our study is unique in the use of these particular tests in population-based non-patient populations to estimate a UPDRS3 score, our results are consistent with these prior studies, suggesting that these motor tests are useful in a variety of study or clinical populations. Nonetheless, validation of our exact predictive model before its application in additional populations is recommended. However, we anticipate the model's usefulness in additional populations, given the model worked well to predict UPDRS3 in two communities with markedly different mean air Mn levels and UPDRS3 scores. While we were only able to explore the potential performance of the model in identifying PD in one of these communities, those results were very encouraging, as well. These findings demonstrate the robust predictive ability of the model in multiple practice settings and further underscore the usefulness of selected motor tasks in screening for parkinsonism.

In summary, using selected accelerometry-based kinematic-UPDRS3 tasks and the grooved pegboard, we developed a UPDRS3 predictive model to identify individuals with potential parkinsonism, which demonstrated good predictive ability. The model performed exceptionally well considering that we applied it in non-patient populations and relied on non-clinicians to administer the tests. These findings have important public health applications in screening for or assessing parkinsonism in clinical or research settings in regions of the world with limited clinical neurologic expertise. The proposed screening tool provides an alternative assessment in these low-resourced environments because of ease of administration.

## Data Availability Statement

Individual participant data that underlie the results reported in this article, after de-identification, will be made available upon reasonable request, following article publication. Data will be made available to investigators whose proposed use of the data has been approved by an independent review committee identified for this purpose and after approval of the protocol by the University of the Witwatersrand Ethics Committee and the Washington University Human Resource Protection Committee. The analysis performed must be solely to achieve aims in the approved proposal. Proposals should be directed to BR, at racetteb@wustl.edu, and requestors will need to sign a data use agreement.

## Ethics Statement

The studies involving human participants were reviewed and approved by Washington University School of Medicine Human Research Protection Office (St. Louis, Missouri, United States) and the University of the Witwatersrand Human Research Ethics Committee (Johannesburg, Gauteng, South Africa). The participants provided their written informed consent to participate in this study.

## Author Contributions

BR, MU, GN, and SN: research project conception, organization, execution, and manuscript preparation review and critique. SN: statistical analysis design. WD and SN: statistical analysis execution. BR, MU, and GN: statistical analysis review and critique. WD: manuscript preparation and writing of the first draft. All authors: approval of final manuscript. All authors contributed to the article and approved the submitted version.

## Conflict of Interest

The authors declare that the research was conducted in the absence of any commercial or financial relationships that could be construed as a potential conflict of interest.

## Abbreviations

AUC: Area under the ROC curve
Mn: Manganese
ROC: Receiver operating characteristic
PD: Parkinson's disease
PM_2.5_: Particulate matter ≤ 2.5
UPDRS3: Unified Parkinson Disease Rating Scale motor subsection 3.
