# Supplementary material for: A Rapid Motor Task-Based Screening Tool for Parkinsonism in Community-Based Studies
Source: Front Neurol. 2021 May 13;12:653066. doi: 10.3389/fneur.2021.653066 (PMC8155367; doi:10.3389/fneur.2021.653066)
Supplement: Supplementary file 1 [file Data_Sheet_1.docx]

**SUPPLEMENTARY MATERIAL**

**Supplement 1: Kinematic data processing and summary measures**

We used Stata version MP 14.2 (StataCorp, College Station, Texas) for kinematic data processing. We created and validated a computer code for processing raw kinematic data.

As in previous kinematic studies (1, 2), in processing, we focused on the angular velocity data. These data are output as the root-mean-square angular velocity in degrees per second. The angular velocities can range from -2000 to 2000°/sec in each of the three axes at each time point, i.e., 768 equidistant time points for each 12-second trial. Traditional kinematic summary measures require only the absolute value of velocity, i.e., the speed. In order to calculate some of the alternative measures we developed, we required both the speed and direction (±) components of velocity. For simplicity and consistency with prior literature, we refer to both angular velocity and angular speed as “velocity” hereafter. We used the direction component to identify when a participant had completed half of each “cycle,” i.e., the upward or downward motion of each finger tap or the clockwise or counterclockwise motion of each hand rotation. Operationally, we identified a half-cycle as a series of adjacent time points with the same rotational direction.

We checked and standardized the data from each trial before calculating the kinematic summary measures. First, we checked for complete sensor failure, i.e., removed the trial if the mean velocity in all axes was <15 °/sec (excepting postural tremor trials). Second, we checked for a complete (approximately 12-second) trial and removed the trial if there were <760 time points (<11.9 seconds). Third, we removed from the trial any time points after the 12-second trial, i.e., any over-recording of movements after the trial ended. Fourth, we removed time points that appeared to be excess movement in the beginning or end of the 12-second sampling period itself. Fifth, we removed pauses at the start or end of the 12-second sampling period. We considered these pauses as a participant’s failure to start the motor task immediately or to continue the task to the end of the sampling period, respectively. We interpreted these pauses as such because they were not associated with a higher Unified Parkinson Disease Rating Scale, motor subsection 3 (UPDRS3) score, which otherwise might indicate that these pauses represented start hesitation or severe decrement, respectively, and therefore would be appropriate to capture as part of the motor assessment. In contrast, we did not remove any pauses in the middle of the sampling period. These mid-sampling pauses were positively associated with UPDRS3 score and therefore likely reflected motor decrement.

Following the above standardization, we calculated several kinematic summary measures (Supplemental Table 1) for each task, hand (dominant, non-dominant), and trial. We calculated these summary measures using all ~768 time points in the appropriate axis (x-axis for finger tapping, y-axis for hand rotation, x-axis for postural tremor, and maximum velocity axis for action tremor). After calculating these summary measures for each task/hand/trial, we calculated the mean of the summary measures across all available trials (≤3) for the specified hand and task. We then compared these to the summary measures derived from only the first trial and determined that a single trial would be sufficient in field-based studies (Supplemental Tables 3 and 7). Finally, we calculated a variation of the mean velocity summary measures in which we determined the velocity according to which part of the movement was being captured, e.g., finger extension vs. finger closing for the finger tapping task (Supplemental Table 1).

Prior to conducting our main analyses, we verified that the mean velocity, mean peak velocity, and coefficient of variation for the finger tapping and hand rotation tasks agreed with the same measures that we calculated through a substantially more labor-intensive method in which we used OriginPro 2017 (OriginLab Corporation, Northampton, Massachusetts). The OriginPro 2017 method makes use of a point-and-click interface alongside a detailed processing protocol. Among participants with the latter measures, 87.4% (264/302), we observed very high agreement between the two processing methods (Spearman’s ρ=0.95 to 0.99).

**Supplement 2: UPDRS3 subscores and calculation of Spearman’s ρ for assessment of the kinematic and grooved pegboard summary measures**

We focused on the following individual upper limb subscores when assessing all of the kinematic and grooved pegboard summary measures: action/postural tremor, finger tapping, hand movements, and rapid alternating movement of hands. We also calculated an overall upper limb bradykinesia score which was the sum of finger tapping, hand movements, and rapid alternating movement of hands subscores. This combined upper limb bradykinesia score provided a single measure that we could use as one gold standard measure for all upper limb bradykinesia tasks and, hence, the ability to obtain fully comparable results across these tasks.

We then estimated Spearman’s ρ correlation coefficients of each of the kinematic and grooved pegboard summary measures and the respective UPDRS3 subscores (our “gold standard”). Spearman’s ρ is the preferred measure of agreement in an inter-method reliability study where it is assumed that the two methods will provide estimates that are correlated, i.e., that the non-gold standard measure is a potential substitute for the gold standard measure. Therefore, the magnitude of Spearman’s ρ is substantially more informative than the associated p-value. Accordingly, although we do report p-values, we primarily report Spearman’s ρ, i.e., the magnitude and direction of the association, with zero indicating no correlation and 1.0 or -1.0 indicating perfect correlation. We also noted whether the direction of the association was as expected, given that the magnitude is only useful if the direction of the association is as expected. When estimating Spearman’s ρ, we retained the kinematic and grooved pegboard summary measures as calculated. We did not translate these summary measures into UPDRS3 clinical subscores of 0-4. This translation would have introduced measurement error, and otherwise not changed the ranking of participants.

Supplementary References

1. Espay AJ, Giuffrida JP, Chen R, Payne M, Mazzella F, Dunn E, et al. Differential response of speed, amplitude, and rhythm to dopaminergic medications in Parkinson's disease. *Mov Disord* (2011) 26:2504-2508.

2. Heldman DA, Giuffrida JP, Chen R, Payne M, Mazzella F, Duker AP, et al. The modified bradykinesia rating scale for Parkinson's disease: reliability and comparison with kinematic measures. *Mov Disord* (2011) 26:1859-1863.

**Supplemental Table 1. Kinematic summary measures**

**^a^** Calculated only for the upper limb bradykinesia tasks because not applicable for the action/postural tremor tasks.

| Kinematic measure | Description | Reason for inclusion |
| --- | --- | --- |
| Mean velocity | Mean of the velocity across all 786 time points in a single 12-second trial. | Overall measure of speed, as calculated by other researchers. |
| Mean peak velocity | Mean of the velocity across the velocity peaks in a single 12-second trial. Each velocity peak was defined as the velocity at the time point in which the maximum velocity is achieved for each half-cycle (and that appeared to be a true maximum, i.e., >250 °/sec). | Approximation of mean peak velocity, which has been included in prior studies. |
| Coefficient of variation | Ratio of the mean to the standard deviation. | Greater values indicate more variability, i.e., less regularity of movement, and therefore is a potential measure of decrement in velocity. Included in prior studies. |
| Decrement in peak velocity^a^ | Slope (β estimate from linear regression) of the change in peak velocity as a function of time over the 12-second trial. | Estimate of the decrement in the peak velocity. |
| Cycles/second | The number of taps or rotations (cycles) in a second. | Frequency of tapping or rotations. |
| Decrement in cycles/second^a^ | Slope (β estimate from linear regression) of the change in the cycles/second as a function of time over the 12-second trial. | Estimate of the decrement in the frequency. |

**Supplemental Table 2. Correlation^a^ between UPDRS3 upper limb bradykinesia subscores and kinematic/grooved pegboard tests**

Abbreviations: UPDRS3=Unified Parkinson’s Disease Rating Scale motor subsection 3. ^a^ Spearman’s ρ. A bolded value indicates p <0.05. ^b^ Based on ≤3 trials per hand for each of 302 participants. ^c^ Greater UPDRS3 scores and subscores indicate greater parkinsonism, which we anticipated would be associated with longer grooved pegboard times, fewer pegs placed, more pegs dropped, and then on the finger tapping and hand rotation tasks slower movement (lower velocities or fewer cycles per second), as well as greater decrement and hence greater variability in the latter. ^d^ Rapid alternating movements, finger taps, and hand movements. ^e^ While the action tremor task was included to assess action tremor, we observed that the mean velocity for this task was correlated with upper limb bradykinesia subscore.

| Test, measure, and  expected direction of association^c^ | Dominant hand  N=302^b^ | | | Non-dominant hand  N=302^b^ | | |
| --- | --- | --- | --- | --- | --- | --- |
|  | **UPDRS3 rapid alternating movements (hand rotation)** | **UPDRS3 finger taps** | **UPDRS3 overall upper limb bradykinesia**^d^ | **UPDRS3 rapid alternating movements**  **(hand rotation)** | **UPDRS3 finger taps** | **UPDRS3 overall upper limb bradykinesia**^d^ |
| Kinematic test: Hand rotation |  |  |  |  |  |  |
| Mean velocity (-) | **-0.43** | N/A | **-0.40** | **-0.42** | N/A | **-0.39** |
| Mean peak velocity (-) | **-0.39** | N/A | **-0.33** | **-0.38** | N/A | **-0.34** |
| Coefficient of variation (+) | **0.37** | N/A | **0.36** | **0.37** | N/A | **0.35** |
| Cycles/second (-) | -0.09 | N/A | -0.08 | -0.06 | N/A | -0.07 |
| Decrement in peak velocity (-) | 0.03 | N/A | 0.04 | -0.04 | N/A | -0.07 |
| Decrement in cycles/second (-) | 0.02 | N/A | 0.0002 | **-0.14** | N/A | **-0.21** |
| Kinematic test: Finger taps |  |  |  |  |  |  |
| Mean velocity (-) | N/A | **-0.30** | **-0.29** | N/A | **-0.26** | **-0.27** |
| Mean peak velocity (-) | N/A | **-0.25** | **-0.25** | N/A | **-0.23** | **-0.22** |
| Coefficient of variation (+) | N/A | **0.21** | **0.19** | N/A | **0.14** | **0.16** |
| Cycles/second (-) | N/A | -0.09 | -0.10 | N/A | **-0.19** | **-0.20** |
| Decrement in peak velocity (-) | N/A | -0.05 | -0.08 | N/A | **-0.14** | **-0.12** |
| Decrement in cycles/second (-) | N/A | -0.04 | -0.06 | N/A | **-0.16** | -0.11 |
| Kinematic test: Action tremor^e^ |  |  |  |  |  |  |
| Mean velocity (-) | **-0.17** | **-0.17** | **-0.20** | **-0.28** | **-0.24** | **-0.32** |
| Mean peak velocity (-) | -0.11 | -0.09 | **-0.14** | **-0.19** | **-0.15** | **-0.22** |
| Coefficient of variation (+) | 0.04 | **0.13** | 0.08 | 0.10 | 0.09 | 0.10 |
| Cycles/second (-) | -0.10 | -0.07 | -0.11 | **-0.11** | -0.06 | -0.09 |
| Decrement in peak velocity (-) | -0.01 | -0.03 | -0.002 | **-0.13** | -0.11 | -0.11 |
| Decrement in cycles/second (-) | 0.08 | 0.05 | 0.10 | 0.01 | 0.06 | 0.06 |
| Grooved pegboard test |  |  |  |  |  |  |
| Time in seconds (+) | **0.28** | **0.15** | **0.25** | **0.29** | **0.20** | **0.28** |
| Number of placed pegs (-) | -0.05 | -0.02 | -0.03 | -0.09 | -0.03 | -0.06 |
| Number of dropped pegs (+) | 0.03 | -0.01 | 0.004 | 0.04 | 0.01 | 0.01 |

**Supplemental Table 3. Correlation^a^ between UPDRS3 action/postural tremor subscores and kinematic tests**

Abbreviations: UPDRS3=Unified Parkinson Disease Rating Scale motor subsection 3. ^a^ Spearman’s ρ. A bolded value indicates p <0.05. ^b^ Based on ≤3 trials per hand for each of 302 participants. ^c^ For action tremor only. For the kinematic data for the action tremor task we took the maximum velocity axis, so summary measures from this motor task likely capture the movement of the hand going between the subject’s nose to the examiner’s finger. A similar movement has been used previously as a measure of upper limb bradykinesia. Greater UPDRS3 scores and subscores indicate greater parkinsonism which we anticipated would be associated with tremor, as well as slower movement (lower velocities or fewer cycles per second), greater decrement and hence greater variability in the latter.

| Test, measure, and  expected direction of association^c^ | Dominant hand  N=302^b^ | Non-dominant hand  N=302^b^ |
| --- | --- | --- |
| Kinematic test: Action tremor |  |  |
| Mean velocity (-) | -0.06 | -0.01 |
| Mean peak velocity (-) | -0.02 | 0.03 |
| Coefficient of variation (+) | 0.08 | 0.07 |
| Cycles/second (-) | 0.06 | 0.05 |
| Decrement in peak velocity (-) | -0.01 | -0.01 |
| Decrement in cycles/second (-) | 0.01 | -0.04 |
| Kinematic test: Postural tremor |  |  |
| Mean velocity | 0.06 | -0.01 |
| Coefficient of variation | -0.11 | -0.06 |

**Supplemental Table 4. Correlation^a^ between UPDRS3 upper limb bradykinesia subscores and kinematic/grooved pegboard tests, first trial only**

Abbreviations: UPDRS3=Unified Parkinson’s Disease Rating Scale motor subsection 3. ^a^ Spearman’s ρ correlation. A bolded value indicates p <0.05. ^b^ Agreement calculated based on 1 trial per hand for each of 275 participants. ^c^ Greater UPDRS3 scores and subscores indicate greater parkinsonism, which we anticipated would be associated with longer grooved pegboard times, fewer pegs placed, more pegs dropped, and then on the finger tapping and hand rotation tasks slower movement (lower velocities or fewer cycles per second), as well as greater decrement and hence greater variability in the latter. ^d^ Rapid alternating movements, finger taps, and hand movements.

| Test, measure, and  expected direction of association^c^ | Dominant hand  N=275^b^ | | | Non-dominant hand  N=275^b^ | | |
| --- | --- | --- | --- | --- | --- | --- |
|  | **UPDRS3 rapid alternating movements** | **UPDRS3 finger taps** | **UPDRS3 overall upper limb bradykinesia**^d^ | **UPDRS3 rapid alternating movements** | **UPDRS3 finger taps** | **UPDRS3 overall upper limb bradykinesia**^d^ |
| Kinematic test: Hand rotation |  |  |  |  |  |  |
| Mean velocity (-) | **-0.41** | N/A | **-0.39** | **-0.39** | N/A | **-0.37** |
| Mean peak velocity (-) | **-0.39** | N/A | **-0.35** | **-0.35** | N/A | **-0.32** |
| Coefficient of variation (+) | **0.28** | N/A | **0.29** | **0.35** | N/A | **0.30** |
| Cycles/second (-) | -0.08 | N/A | -0.09 | -0.05 | N/A | -0.06 |
| Decrement in peak velocity (-) | 0.05 | N/A | 0.08 | -0.03 | N/A | -0.06 |
| Decrement in cycles/second (-) | 0.09 | N/A | 0.11 | 0.004 | N/A | -0.05 |
| Kinematic test: Finger taps |  |  |  |  |  |  |
| Mean velocity (-) | N/A | **-0.28** | **-0.26** | N/A | **-0.32** | **-0.31** |
| Mean peak velocity (-) | N/A | **-0.26** | **-0.27** | N/A | **-0.30** | **-0.29** |
| Coefficient of variation (+) | N/A | **0.18** | **0.14** | N/A | 0.11 | **0.13** |
| Cycles/second (-) | N/A | -0.09 | -0.09 | N/A | **-0.14** | **-0.15** |
| Decrement in peak velocity (-) | N/A | -0.01 | -0.03 | N/A | 0.003 | 0.01 |
| Decrement in cycles/second (-) | N/A | 0.03 | 0.02 | N/A | -0.03 | -0.04 |
| Kinematic test: Action tremor |  |  |  |  |  |  |
| Mean velocity (-) | **-0.14** | **-0.12** | **-0.15** | **-0.30** | **-0.25** | **-0.33** |
| Mean peak velocity (-) | -0.04 | -0.04 | -0.06 | **-0.22** | **-0.17** | **-0.23** |
| Coefficient of variation (+) | 0.09 | 0.10 | 0.08 | 0.04 | 0.03 | 0.04 |
| Cycles/second (-) | -0.06 | -0.10 | -0.12 | **-0.13** | -0.08 | -0.11 |
| Decrement in peak velocity (-) | 0.01 | -0.01 | 0.01 | 0.001 | 0.03 | 0.02 |
| Decrement in cycles/second (-) | **0.15** | **0.12** | **0.16** | 0.01 | 0.05 | 0.04 |
| Grooved pegboard test |  |  |  |  |  |  |
| Time in seconds (+) | **0.29** | **0.15** | **0.26** | **0.31** | **0.17** | **0.27** |
| Number of placed pegs (-) | **-0.13** | -0.11 | **-0.14** | **-0.13** | -0.06 | -0.11 |
| Number of dropped pegs (+) | 0.04 | 0.02 | 0.03 | 0.06 | 0.04 | 0.05 |

**Supplemental Table 5. Percent sensitivity, specificity, and classified correctly by the UPDRS3 predictive model in the test dataset (N=40), by selected cut points for predicted and neurologist assessed UPDRS3, South Africa, 2016-2020**

Abbreviations: UPDRS3=Unified Parkinson’s Disease Rating Scale motor subsection 3. ^a^ Predicted UPDRS3 score cut points are rounded to the nearest integer. Only cut points with minimal (<5%) rounding error and with materially different sensitivity, specificity, and/or classified correctly than other cut points are shown.

|  | Neurologist assessed UPDRS3 | | | | | |
| --- | --- | --- | --- | --- | --- | --- |
|  | **UPDRS3 ≥10** | | | **UPDRS3 ≥15** | | |
| Predicted UPDRS3^a^ | **Sensitivity** | **Specificity** | **Classified correctly** | **Sensitivity** | **Specificity** | **Classified correctly** |
| ≥ 5 | 100.0 | 25.0 | 62.5 | 100.0 | 14.7 | 27.5 |
| ≥ 6 | 95.0 | 40.0 | 67.5 | 100.0 | 26.5 | 37.5 |
| ≥ 7 | 85.0 | 50.0 | 67.5 | 100.0 | 38.2 | 47.5 |
| ≥ 8 | 70.0 | 70.0 | 70.0 | 100.0 | 58.8 | 65.0 |
| ≥ 9 | 65.0 | 80.0 | 72.5 | 100.0 | 67.7 | 72.5 |
| ≥ 11 | 50.0 | 90.0 | 70.0 | 83.3 | 79.4 | 80.0 |
| ≥ 13 | 40.0 | 95.0 | 67.5 | 66.7 | 85.3 | 82.5 |
| ≥ 14 | 30.0 | 95.0 | 62.5 | 66.7 | 91.2 | 87.5 |
| ≥ 16 | 20.0 | 100.0 | 60.0 | 50.0 | 97.1 | 90.0 |
| ≥ 17 | 15.0 | 100.0 | 57.5 | 50.0 | 100.0 | 92.5 |

**Supplemental Table 6. Percent sensitivity, specificity, and classified correctly by the UPDRS3 predictive model in the external validation dataset (N=90), by selected cut points for predicted UPDRS3, South Africa, 2016-2020**

Abbreviations: UPDRS3=Unified Parkinson’s Disease Rating Scale motor subsection 3. ^a^ Predicted UPDRS3 score cut points are rounded to the nearest integer. Only cut points with minimal (<5%) rounding error and with materially different sensitivity, specificity, and/or classified correctly than other cut points are shown. ^b^ When allowing non-integer cut points sensitivity remained at 100.0% while specificity increased to 67.1% when using a cut point of 3.898.

|  | Neurologist Assessed UPDRS3 ≥10 | | |
| --- | --- | --- | --- |
| Predicted UPDRS3^a^ | **Sensitivity** | **Specificity** | **Classified correctly** |
| ≥ 3 | 100.0% | 47.6%^b^ | 51.1% |
| ≥ 4 | 83.3% | 68.3% | 69.3% |
| ≥ 5 | 66.7% | 78.1% | 77.3% |
| ≥ 6 | 50.0% | 86.6% | 84.1% |
| ≥ 7 | 33.3% | 91.5% | 87.5% |
| ≥ 11 | 33.3% | 100.0% | 95.5% |


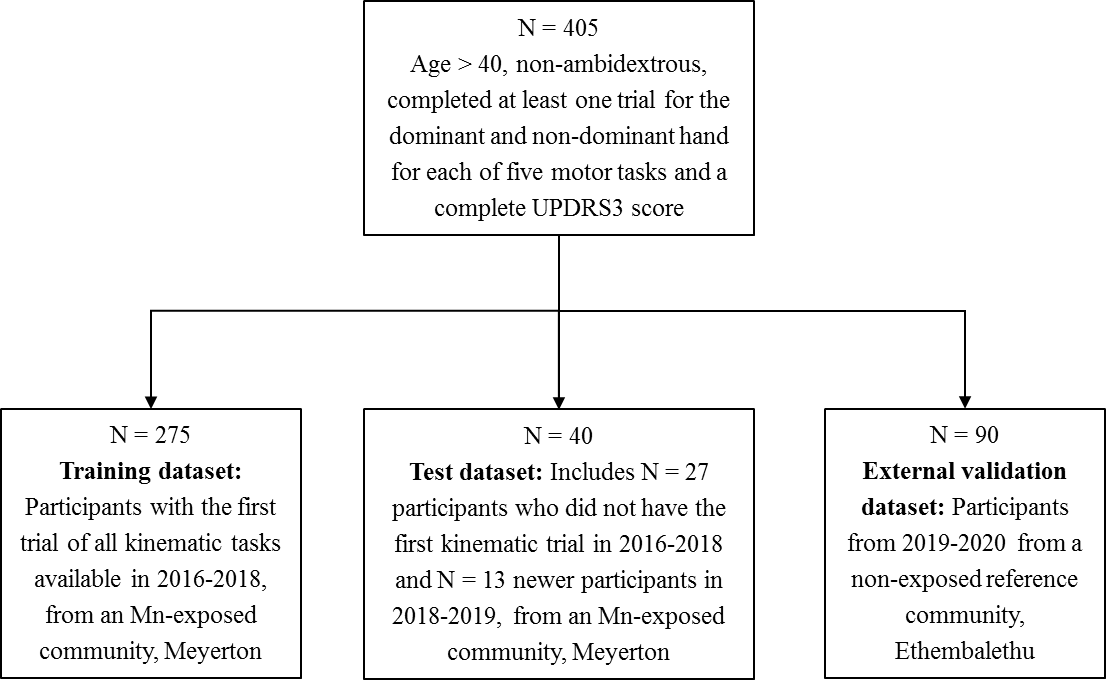


**Supplemental Figure 1:** UPDRS3 predictive model sample, South Africa, 2016-2020. Participating Meyerton residents were older than age 40 years and had complete grooved pegboard and kinematic data for the required trial(s) and a complete UPDRS3 score.

Abbreviations: UPDRS3=Unified Parkinson’s Disease Rating Scale motor subsection 3.


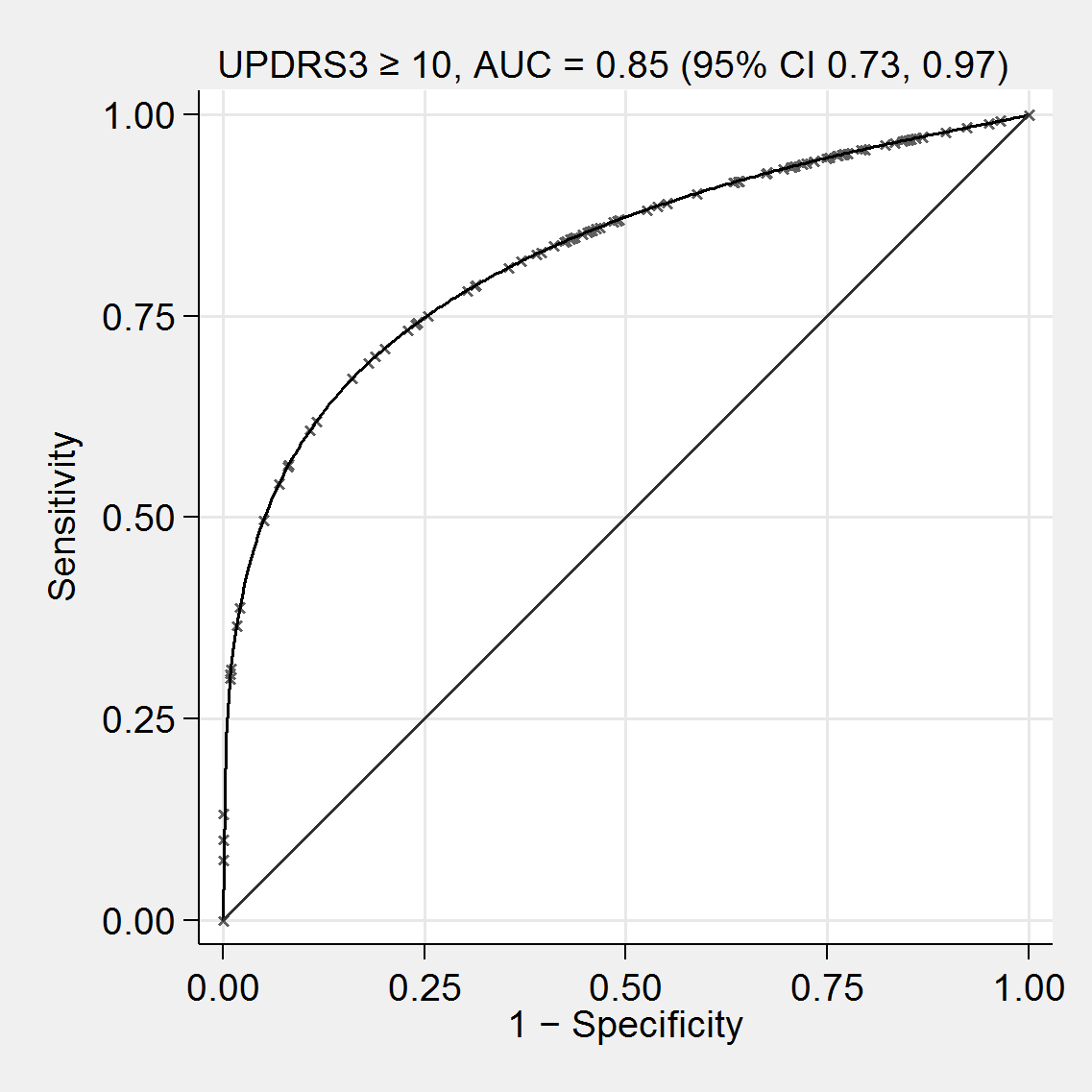


**Supplemental Figure 2:** Receiver operating characteristic curve for UPDRS3 ≥10 in a population-based sample without excess environmental Mn exposure, South Africa, 2016-2020. The AUC indicates that the UPDRS3 predictive model performed well.

Abbreviations: AUC=area under the receiver operating characteristic curve; UPDRS3=Unified Parkinson’s Disease Rating Scale motor subsection 3; Mn=manganese.
